# Using Machine Learning for the Automated Segmentation and Detection of Swallows Obtained by Digital Cervical Auscultation in Preterm Neonates

**DOI:** 10.1007/s00455-025-10879-3

**Published:** 2025-09-12

**Authors:** Stephen So, Timothy Tadj, Belinda Schwerin, Anne B. Chang, Seiji Humphries, Thuy T. Frakking

**Affiliations:** 1 Research Development Unit, Caboolture Hospital, Metro North Hospital & Health Service, McKean St, Caboolture, QLD 4510 Australia; 2 Child Health Research Centre, School of Medicine, The University of Queensland, South Brisbane, QLD 4101 Australia; 3 Speech Pathology Department, Gold Coast University Hospital, Gold Coast Hospital & Health Service, 1 Hospital Boulevard, Southport, QLD 4215 Australia; 4 School of Health Sciences & Social Work, Griffith University, 1 Parklands Drive, Southport, Gold Coast, QLD 4222 Australia; 5 School of Engineering and Built Environment, Griffith University, Parklands Dr, Southport, QLD 4215 Australia; 6 Department of Respiratory Medicine, Queensland Children’s Hospital, 501 Stanley St,, South Brisbane, QLD 4101 Australia; 7 Child and Maternal Health Division, Menzies School of Health Research, Charles Darwin University, PO Box 41096, Casuarina, NT 0811 Australia; 8 Australian Centre for Health Services Innovation, Queensland University of Technology, Level 7, 62 Graham St,, South Brisbane, QLD 4101 Australia

**Keywords:** Cervical auscultation, Deglutition, Swallowing sounds, Signal processing, Machine learning, Preterm, Neonate

## Abstract

The clinical application of acoustic swallowing sound parameters collected from digital cervical auscultation is limited because of the time-consuming manual segmentation required by trained experts. The automated identification of swallowing sounds in children and adults from swallowing sound audio wavefiles using machine learning have accuracies between 76–95%. No data exists in preterm neonates. To determine if applying automated machine learning using a transfer learning approach could accurately identify and segment swallows from swallowing sounds collected in preterm neonates. Thin fluid swallow sounds were collected from 78 preterm neonates, median birth age 34 weeks gestation (range 25–36 weeks, 52.6% males) across 3 Australian special care nurseries. For the base machine learning model, a deep convolutional neural network (DCNN) pre-trained for audio event classification was used. With raw swallow audio data as input, embedding vectors from the base DCNN were generated and used to train a feedforward neural network to determine the presence of a swallow within an audio segment. The model showed high overall accuracy (94%) in identifying preterm swallows. Better model performance on bottle feeding swallows (Sensitivity, 95%; and specificity, 96%) was seen compared with breastfeeding swallows (sensitivity, 95%, specificity 92%). Interpretation: Our novel study demonstrates the successful use of transfer learning to accurately identify and segment digital swallowing sounds in preterm neonates. Application of this model could support the development of a digital CA app to automatically classify swallow sounds and improve objectivity for CA use in clinical practice within special care nurseries.

**Supplementary Information:**

The online version contains supplementary material available at 10.1007/s00455-025-10879-3.

## Introduction

The prevalence of feeding difficulties in preterm neonates is estimated to be 10%, with higher prevalence rates in very low birth weight neonates (< 1500 g) or those with chronic neonatal lung disease (24–70%) [1, 2]. Oropharyngeal aspiration (abbreviated to aspiration) is defined as the entry of fluids into the trachea below the level of the vocal cords. Aspiration occurs in 40–50% of preterm neonates with feeding difficulties. Importantly, 80% of preterm neonates with feeding difficulties have silent aspiration and do not show overt clinical signs of aspiration (e.g. cough) [2, 3]. Undiagnosed aspiration can lead to acute and chronic lung sequelae (e.g. pneumonia, chronic cough, damage to the developing lungs, increasing the severity of chronic neonatal lung disease) [4, 5]. In addition, the impact of feeding difficulties in preterm neonates often results in the delayed transition from tube to full oral feeding. Up to 40% of preterm neonates are discharged on tube feeding, and delayed transition from tube to full oral feeding often results in a longer hospital stay [6]. Reliance on tube feeding costs the healthcare system approximately $46 875 for the first year in supplies and management of feeding tubes and $180,000 per child aged > 5 years [7].

The assessment and management of aspiration in preterm neonates is complex, given the evolving nature of underlying pathophysiology, maturing neurosensory/neuromotor processes, heterogeneity of clinical presentations and reliance of multidisciplinary healthcare professionals [8]. In current clinical practice, a videofluoroscopic swallow study (VFSS) is the standard reference method for diagnosis and assessment of aspiration. It allows visualisation of all phases of the swallow, including the direct visualisation of aspiration events [9]. However, the use of VFSS is limited by the cumulative exposure to ionizing radiation after repeat assessments [10, 11]. Feeding performances at each attempt on oral feeding can be variable in preterm neonates, making a single VFSS difficult to interpret [12]. VFSS may not replicate typical feeding performances due to the time constraints associated with minimisation of screening time and difficulties replicating typical infant formula with barium impregnated fluids [13, 14]. VFSS is also limited by accessibility due to the high costs associated with medical imaging equipment and the requirement of multiple health professionals specifically trained in conducting and interpreting paediatric VFSS [13]. Thus, complementary assessment methods for the identification of swallowing and aspiration in infants and children should be explored.

Cervical auscultation (CA), is a popular adjuvant to the clinical feeding examination which relies on listening to swallow and breath sounds pre- and post-swallow [15]. Using VFSS as the gold standard, diagnostic test accuracy studies have demonstrated that digital CA has high sensitivity 0.85 (95%CI 0.62–0.97), high negative predictor value 0.92 (95%CI 0.78–0.98), very good inter-rater (0.81; 95%CI 0.79–0.84) and good to very good intra-rater (kappa range 0.72–0.98) reliability in detecting aspirating swallows in term neonates and children with paediatric feeding disorders [16, 17]. To our knowledge, no diagnostic test accuracy studies exist on CA within the preterm neonatal population. This is a critical gap in clinical practice and the literature, given known differences in the oromotor feeding skills of preterm and term neonates in relation to sucking and swallowing rates, length and efficiency of suck bursts, and swallowing function [18, 19]. In addition, specific preterm factors such as reduced aerodigestive tract size, increased respiratory rates, underlying etiology (e.g. laryngomalacia, chronic neonatal lung disease), reduced muscle tone and fatty tissue in the oropharynx have the potential to influence swallowing and breath sound parameter [20, 21].

In the past decade, there have been improvements to the objectivity of CA in adults and children where classifiers can differentiate between impaired and non-impaired swallows; and CA sound features have correlated to swallow kinematic events such as hyoid bone displacement, laryngeal closure and upper oesophageal sphincter opening [22–33]. In contrast, limited studies of CA in preterm neonates exist. Seminal work by Reynolds and colleagues demonstrated that the variance index (based on swallowing sound waveforms) increased in signal consistency as preterm neonates reached term age; and that there were differences in swallowing sound waveforms between preterm neonates with and without chronic neonatal lung disease [34, 35]. However, both studies were limited by extremely small sample sizes (n = 10 & 17, respectively), used inferior audio recording technology compared to technologies presently available and lacked objective assessments (e.g. VFSS). The advancements in the objectivity of digital CA have so far relied on the manual segmentation of swallows by a trained expert, which is labour intensive and a key barrier to the translation of digital CA into routine practice. Application of machine learning to automate the detection and segmentation of swallows could help: (a) facilitate the use of digital CA in the assessment of swallowing in the preterm population, (b) support the development of a digital CA app that can objectively classify swallow sounds and improve objectivity for use in clinical practice; and (c) eliminate the need for time consuming manual segmentation of swallow sounds by clinicians/researchers with experience in CA.

A range of works have investigated the automatic detection of swallowing sounds, however these are typically difficult to comparatively evaluate and validate due to a lack of standardized approach [36]. Works can generally be grouped into those utilizing microphone sound recordings of swallows, and most prevalently, those segmenting accelerometry recordings. For the later, Khalifa and colleagues used segmented spectrograms as into to a Deep Neural Network (DNN), reporting 89% average accuracy on clean swallows and 76% across the whole dataset of accelerometer recordings on adult participants [37]. However, accelerometry recordings are not presently accessible by clinicians, making the translation to practice impractical. Those investigating the segmentation of swallowing sounds recorded via microphone commonly apply methods founded in speech processing and automatic speech recognition. Approaches include those using thresholding of either acoustic or spectral features, Mel Frequency Cepstrum Coefficients (MFCC) in Hidden Markov Models (HMM), MFCCs and variations of these in Gaussian Mixture Model (GMM), Mel-scale Fourier spectral features in a support vector machine (SVM), and dynamic time-warping template matching in the frequency domain [38–43]. Kimura and colleagues used a variety of acoustic features of automatic detection of swallow sounds, reporting an accuracy of 95.2% [44]. More recently, Neural Network and Deep Learning based approaches have been proposed. Aboofazeli and colleagues used spectral features as input to a two layer feedforward Neural Network (NN) on a small data set of tracheal sound recordings from healthy adult participants, reporting a 91.7% average accuracy [45]. Works surveyed predominately considered adult, either healthy or with specific conditions, rather than pediatric participants, and had mixed experimental conditions (e.g. saliva, fluid and food swallows). Further, the labelling of the presence or absence of swallowing sounds relied on manually determined labels, rather than use of more objective measures such as the VFSS.

Our study presents a machine learning method for automatic segmentation and detection of swallow and non-swallow sounds from digital cervical auscultation recordings of preterm neonates, thereby enabling start and end points of each swallow to be detected with high accuracy. Please see Supplemental Table 1 for a glossary for digital signal processing and artificial intelligence terms. As training deep neural network models requires very large quantities of human-labelled data to avoid low generalisation performance and overfitting, and gathering such large volumes of swallow data is impractical, transfer learning was utilised. Transfer learning uses deep learning models trained on large amounts of related data, to enhance performance in a similar classification task with limited amounts of labelled data. In this way information learnt on the previous task is reused to help boost performance on the new task. In the proposed model, the publicly available YAMNet (Yet another Audio Mobilenet Network) model for audio classification was used [46]. YAMNet is a deep convolutional neural network that has been pre-trained to predict 521 different types of audio events from 2,084,320 human-labelled 10 s audio clips (AudioSet dataset) from YouTube videos. Sounds in the dataset include musical instruments, human and animal sounds, and everyday environment sounds [47]. The acoustic features and embeddings learnt in YAMNet for discriminating the wide range of audio classes, then help identify distinct audio events associated with swallowing from other environment sounds. Thus, in this study aimed todetermine if applying automated machine learning using a transfer learning approach could accurately identify and segment swallows from swallowing sounds collected in preterm neonates. Given that transfer leaning allows adaptation of an existing neural network model which has been previously trained on very large quantities of data and similar category of machine learning task, we hypothesized that this approach is able to assist with discriminating audio classes such as those associated with identifying swallowing sounds.

## Methods

This prospective cross-sectional study received relevant Human Research Ethics Committee (HREC) approvals across Queensland Health (HREC/2021/QRBW/76999) and Universities (2022/HE000128 and GU Ref No: 2022/129).

### Participants

Random sampling recruitment of 78 preterm neonates across 3 Australian hospitals with level 6 (Royal Brisbane & Women’s Hospital and Gold Coast University Hospital) and level 4 (Caboolture Hospital) clinical services capability was completed between October 2021 to July 2022. Swallowing sounds were collected from feeding observations. Inclusion criteria were: ≤ 36^+6^weeks and demonstrated ability to tolerate bolus feeds and rating of 1 or 2 score on the infant-driven feeding readiness scale at oral feeding times [48]. Exclusion criteria were: the presence of any structural (e.g. cleft lip or palate) or chromosomal abnormality that affected feeding/swallowing ability. Preterm neonates had a range of medical co-morbidities which included the following most common conditions: low to very low birth weight, jaundice, apnoea of prematurity, hypoglycaemia, respiratory distress syndrome and retinopathy of prematurity.

### Procedure

Two minute simultaneous audio-visual recordings of from feeding observations of preterm neonates breast or bottle feeding were collected. All preterm neonates were fed in standard feeding positions (e.g. elevated side lying, semi-reclined position) by their caregiver or treating nurse.

### Equipment

**Fig. 1:**
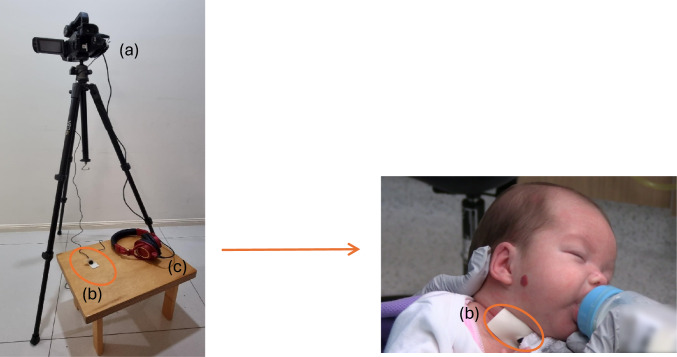
Equipment set up includes **a** digital video recorder (Model XA40, Cannon Incorporation, Tokyo, Japan); **b** microphone was inserted into a fitted O-ring (C417, AKG Acoustics, Vienna, Austria); and **c** Headphones (Model ATH-M50x, Audio-Technica, Taiwan)

Simultaneous audio-visual recordings were collected using an omnidirectional condenser microphone (C417, AKG Acoustics, Vienna, Austria) (sensitivity at 1 kHz of 10 mV/Pa, impedance 200Ω, frequency range 20 to 20,000 Hz) directly connected to a digital video recorder (Model XA40, Cannon Incorporation, Tokyo, Japan). The microphone was inserted into a fitted O-ring and attached securely using microfoam tape onto the skin surface of the neonate’s neck, which was immediately lateral to midline. Headphones (Model ATH-M50x, Audio-Technica, Taiwan) worn by researcher TTF were simultaneously connected to the digital video recorder throughout the feeding observation to allow TTF access to the live sound recordings. See Fig. 1. This ensured that the sound recordings could be monitored for quality and microphone re-positioning could be made, as required. For example, reduced sound quality as a result of a reduction between microphone and skin contact.

### Manual Segmentation of Swallow Sounds

Using an acoustic editing software package (Adobe Audition CS6 v5, Adobe Systems, USA), the raw acoustic signal was displayed as a waveform plot (y-axis (amplitude, decibels, dB) versus x-axis (time, seconds, s) (44,100 Hz sampling rate) simultaneously with the visual recording of the neonate breast or bottle feeding and a spectrogram (y-axis: frequency, Hz; x-axis: time, s). The waveform plot was magnified so that a 1-s section of the audio waveform filled the majority of the program’s viewing window. The start and end of each individual swallow was characterized simultaneously with the audio feedback of the swallowing sound (i.e. presence of a fluid flushing sound immediately after the cessation of inspiratory/expiratory respiratory sounds), visualization of the swallow from the visual recording (i.e. wide jaw excursion) and visualization of the spectrogram (i.e. areas of intensity). See supplemental Fig. 1.

The timepoints for swallow sounds were manually segmented by two researchers: TTF, a speech pathologist with 18 years clinical experience in pediatric feeding disorders and postgraduate doctoral skills in pediatric cervical auscultation; and SH, a PhD-qualified health researcher trained by TTF in the identification of swallow sounds from audiofiles using pre-existing pediatric swallow sound files (N = 20). Initially, TTF and SH completed the manual segmentation of swallows for five preterm neonates together. Following this, SH completed the manual segmentation of swallows for the remaining cohort. All manual segmentation data points for the swallow sounds of preterm neonates in this study were reviewed by TTF. Clarification between raters for 21% (722/3355) of swallows for confirmation on the: (a) start and end points of swallows; and (b) differentiation between a nutritive swallow versus extraneous noises (e.g. sucking, non-nutritive swallows) occurred through discussion and achievement of a consensus value between the two raters. Both raters were blinded and independent of model results and were only involved in the manual segmentation stage.

### Data Preprocessing and Feature Extraction Stage

**Fig. 2:**
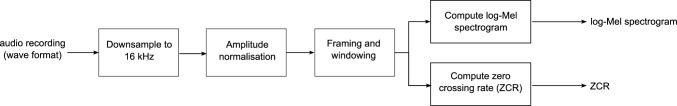
Block diagram of the audio feature extraction stage

Two experienced electrical and electronic engineers (SS and BS) led the data preprocessing and feature extraction stage, and transfer learning and development of the final network model. As shown in Fig. 2, in order to match the sampling rate used in the YAMNet model, the digital audio files were downsampled from 44.1 kHz to 16 kHz using the libsoxr library[49], which uses appropriate signal processing techniques such as interpolation and decimation as well as Kaiser window FIR (or finite impulse response) interpolation filtering. The audio data was then normalised so that the amplitude values occurred between –1 and 1. Please refer to Supplemental Table 1 for a full list of artificial intelligence and signal processing terminology and definitions.

The audio data was then split into frames of 0.96 s with a 50% overlap. Each frame of audio data was converted into a log-Mel spectrogram and these were used as the input audio features to the pre-trained YAMNet model. The mel-scale warping of the frequency is a speech signal processing method that is used to simulate the non-linear frequency resolution of the inner human ear, while the logarithmic operation on the Mel-scale filter outputs simulates the amplitude compression applied by the middle ear. These two operations have been known to increase the robustness of the features to noise in automatic speech recognition.

Complementing the log-Mel spectrogram features, the zero crossing rate (ZCR) was computed for each frame as an additional feature. The ZCR measures the rate at which the waveform changes its sign (i.e. crosses the zero axis) and has been used as a feature in automatic speech recognition [50]. It was found from experimentation that including the ZCR feature significantly improved the segmentation performance of the model.

### Transfer Learning and the Final Neural Network Model

The original YAMNet model consists of multiple convolution layers for feature extraction that are then fed into the top-most softmax classification layer. In the transfer learning approach used in this study, the YAMNet embeddings (i.e. the outputs from the top-most convolution layer that feed into the softmax layer) were computed for each data frame. These embeddings were then concatenated with the ZCR computed for that frame. The softmax layer of the YAMNet model was then replaced with a fully-connected neural network (FCNN) that contained four hidden layers with 1024, 1024, 1024, 512 units, respectively, and an output layer of six units. The rectified linear unit (or ReLU) activation function was used in the input and hidden layers of the FCNN while the sigmoid activation function was used in the output layer. In order to mitigate model overfitting, L2 regularisation was applied to the first three hidden layers.

The participants in the dataset (n = 78) were randomly divided into training, validation, and testing subsets in a 60/20/20 ratio, before the audio data in the training set were balanced and shuffled prior to each training epoch to ensure an equal proportion of swallowing and non-swallowing sounds. The training process adopted in this study used mini-batches of size 32 and utilised the Adam optimiser with the mean squared error (or MSE) used as the loss function. Only the weights in the FCNN were adjusted during the training. A maximum of 70 epochs was set for the training and an early stopping mechanism was set for five epochs to mitigate the effects of overfitting.

**Fig. 3:**
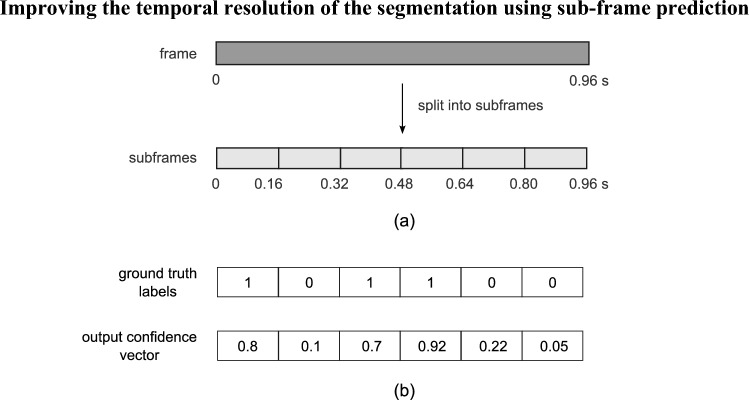
Increasing temporal resolution of segmentation. **a** Each 0.96 s frame is split into six subframes of 0.16 s duration; **b** example of a ground truth label and model output confidence vector showing swallows occurring in subframes 1, 3 and 4

**Fig. 4:**
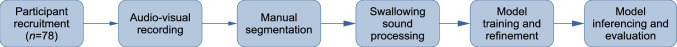
Flow diagram of the methodology employed in this study

The long frame length (0.96 s) used in the original YAMNet was found to be less than ideal for segmentation purposes, since it resulted in poor temporal resolution of the segmentation output. This was identified as a limitation since individual swallows can be relatively short in duration, by comparison, and multiple successive swallows can occur within the long 0.96 s frame. As shown in Fig. 3a, in order to increase the temporal resolution of the segmentation, each long frame was further subdivided into six smaller sub-frames, each of 0.16 s in duration. In the ground truth label (Fig. 3b), the occurrence of a swallow in each subframe was represented by a 1, while non-swallows were assigned the value of 0. The six-unit output layer of the FCNN produced a vector of six confidence values, where any confidence above 0.5 was marked as a predicted swallow. Through this subframe prediction mechanism, the temporal resolution improved by a factor of six, i.e. it was able to detect swallowing events that occurred within a window of 0.16 s. Figure 4 shows a flow diagram of the methodology used in this study.

### Sample Size and Statistical Analyses

We planned for a sample size of 79 preterm neonates, which provides 80% power (two-sided α = 0.05) to detect correlations of at least 0.31 difference between variables [51]. Accuracy in swallow detection was percent agreement between clinician and model ratings. Other estimates of diagnostic accuracy included the following (where TP, FP, TN, FN are the number of true positives, false positives, true negatives, and false negatives, respectively):

- accuracy, which is the proportion of the predictions (positive and negative) that were correct;
- sensitivity (recall), which expresses the proportion of the true swallows that were correctly predicted by the model;
- specificity, which expresses the proportion of the true non-swallowing sounds that were correctly predicted by the model;
- positive (precision) and negative predictive values, which express the proportion of the model’s positive or negative predictions that were correct; and
- F_1_ score, which is defined as the harmonic mean of the precision and recall, is another measure of accuracy that is more conservative, balances the contribution of false negatives and false positives to the final metric, and is better suited to cases where classes are unbalanced [52].

## Results

### Demographics of Participants

The median birth age of the participants was 34 weeks gestation (range 25–36 weeks, 52.6% males), with a median of 36 weeks gestation (range 34–43 weeks) at the time of the feeding observation. The most common medical conditions included: retinopathy of prematurity (66.7%), jaundice (65.4%), hypoglycaemia (43.6%), low birth weight (41%) and apnea of prematurity (23.1%). No serious adverse events occurred in the collection of swallow sounds in our preterm cohort.

### Overall Machine Learning Performance for All Preterm Swallows

A total number of 3355 swallows were used across the training, validation and testing phases of the model. Table 1 lists the overall performance of the model, where the total accuracy was found to be 94%.

**Table 1:** Model performance between patients with non-swallow and swallows (total accuracy = 0.94)

	PPV or Precision	NPV	Specificity	Sensitivity or Recall	F_1_-score
Non-swallow (0)	0.98	0.85	0.95	0.93	0.95
Swallow (1)	0.85	0.98	0.93	0.95	0.90

PPV = positive predictive value; NPV = negative predictive value. The F_1_ score, which is defined as the harmonic mean of the precision and recall, is another measure of accuracy that is more conservative, balances the contribution of false negatives and false positives to the final metric, and is better suited to cases where classes are unbalanced

### Machine Learning Performance for Bottle and Breastfeeding Swallows in Preterms

**Fig. 5:**
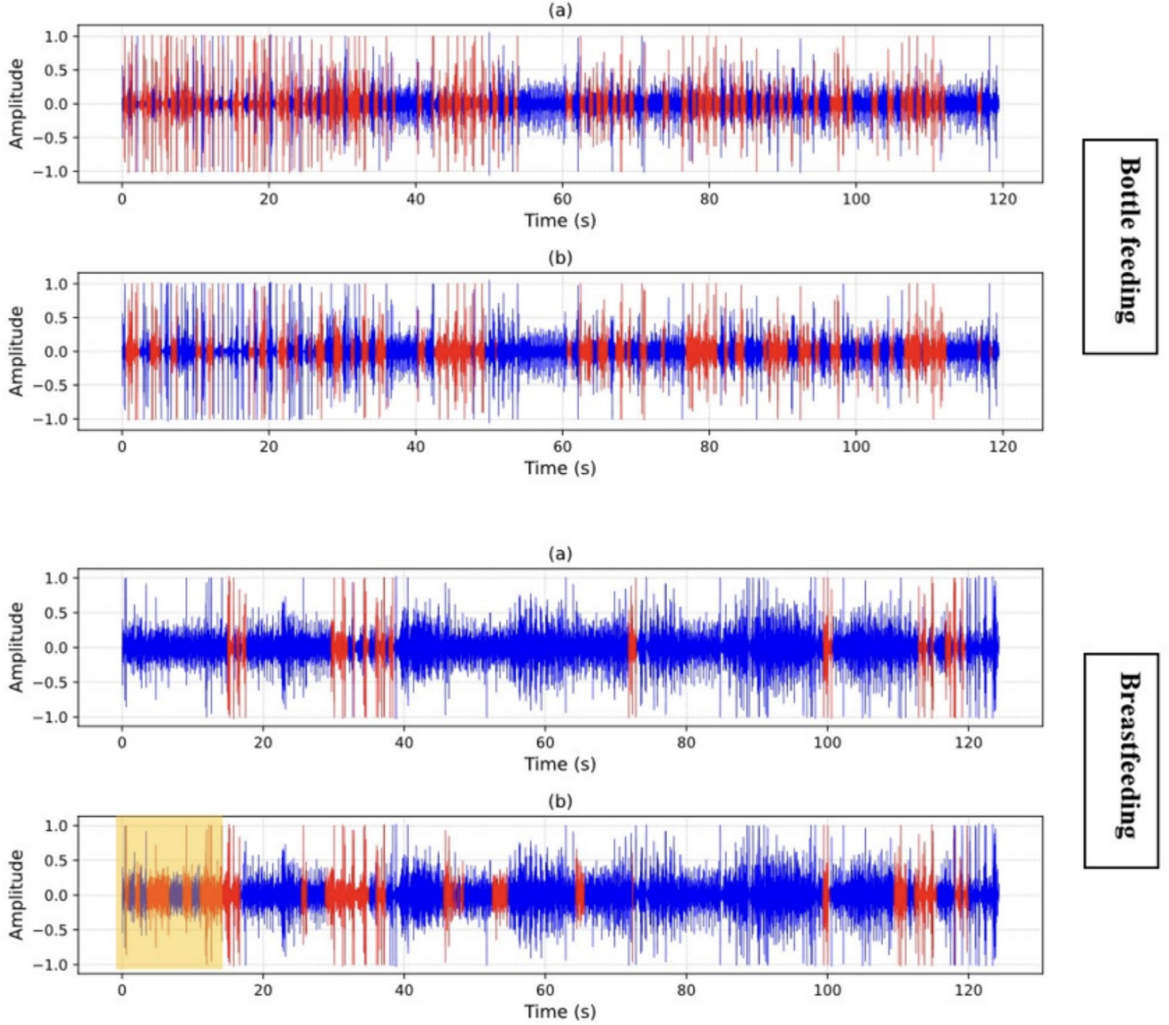
Waveforms showing in red colour the locations of **a** true labelled swallows; and **b** model-predicted swallows of bottle and breastfeeding preterms in the testing dataset. The blue colours represent non-swallowing sections.The yellow shaded region shows possible prediction of non-nutritive swallows by the model

The total accuracy for detecting swallows and non-swallows was higher for bottle fed, compared to breastfeeding swallow sounds. See Table 2 for full diagnostic test accuracy results and Fig. 5 for examples of breast and bottle feeding swallowing sound waveforms.

**Table 2:** Model performance between bottle and breastfeeding preterms with non-swallow and swallows

		PPV or Precision	NPV	Specificity	Sensitivity or Recall	F_1_-score	Total accuracy
Bottle feeding	Non-swallow (0)	0.97	0.94	0.95	0.96	0.97	0.96
	Swallow (1)	0.94	0.97	0.96	0.95	0.94	
Breast feeding	Non-swallow (0)	0.99	0.77	0.95	0.92	0.92	0.93
	Swallow (1)	0.77	0.99	0.92	0.95	0.85	

PPV = positive predictive value; NPV = negative predictive value. The F_1_ score, which is defined as the harmonic mean of the precision and recall, is another measure of accuracy that is more conservative, balances the contribution of false negatives and false positives to the final metric, and is better suited to cases where classes are unbalanced

## Discussion

In this study, we used transfer learning to build a neural network that automatically identifies and segments preterm neonatal swallowing sounds. The model showed high overall accuracy (94%) in identifying preterm swallows, with better model performance on bottle feeding swallows. Transfer leaning allows adaptation of an existing neural network model which has been previously trained on very large quantities of data and similar category of machine learning task. The YAMNet model, which is trained to classify audio from YouTube videos, was selected because it would have learnt higher level representations from input speech recognition features (i.e. Mel-scale-warped spectrograms) in its convolutional network layers. This likely assists with discriminating audio classes such as those associated with identifying swallowing sounds. Therefore, the “learnt” higher level features were transferred to the swallowing segmentation task as inputs to a custom feedforward neural network, which was then trained for the task of identifying swallowing sounds. Using this approach, we were able to overcome the large data requirement to train a deep neural network. The overall high accuracy achieved using our model (94%), with sensitivities of 95% and 93%, for swallowing and non-swallowing sounds, respectively, validates our approach.

In our study, the overall accuracy for the detection and automatic segmentation of swallow sounds was higher in bottle than breastfed preterm infants. It is well known that non-nutritive sucking (NNS) consistently occurs prior to the milk ejection reflex in breastfeeding infants [53]. During this period of NNS, there is minimal milk available until the milk ejection reflex occurs. In contrast, bottle-fed infants have milk flow in their oral cavity as soon as they suck the teat and this leads to nutritive bolus swallows. It is possible that higher rates of NNS in breastfed preterm infants led to increased dry saliva swallows compared to bottle-fed preterm infants who demonstrated consistently more nutritive bolus swallows. The machine learning detection of dry saliva swallows in breastfed infants may have led to false positives because this type of swallow was not manually segmented and classified in the training and validation datasets. In adults, dry saliva and bolus swallows are similar in the location of brain activation (e.g. pre-central motor and post-central somatosensory cortical regions), have similar swallow apnoea durations and both generate pharyngeal and upper oesophageal sphincter pressures that can influence swallow sound acoustic characteristics of amplitude (loudness) and duration (length) [54–57]. Additionally, preterm infants have increased lingual displacement and excursion patterns during nutritive sucking compared in NNS [58]. We hypothesize that this leads to larger volumes of milk and more audible nutritive swallows during bottle feeding that facilitates accurate machine learning detection compared to quieter NNS and dry saliva swallows seen in breastfed preterm infants. Despite these challenges with accurately categorising NNS versus nutritive swallows in breastfeeding, the model was still able to identify nutritive swallows to a very high degree (93%).

## Limitations

A limitation of this study is the absence of instrumental assessment (e.g. VSS) to objectively identify the start and end point of the preterm swallowing sounds. However, it would unethical to conduct a VFSS and expose the preterm neonates enrolled in this study to radiation given there were no concerns with their feeding/swallowing skills. Future studies could consider concurrently collecting swallowing sounds with Fiberoptic Endoscopic Evaluation of Swallowing (FEES) to mitigate concerns for radiation exposure. FEES is well tolerated in the neonatal population despite being an invasive procedure [59, 60]. Additionally, inter- and intra-rater reliability was not completed and this may compromise the internal validity of our results despite two raters used to manually segment the preterm swallowing sounds. However, rater TTF has received post-doctoral training in cervical auscultation where structural training has been found to increase the validity and reliability of detecting dysphagic swallows [61]. A previous study has shown that rating cervical auscultation sounds collected in children had high inter- and intra-rater reliability among a group of speech-language pathologists at a pediatric tertiary hospital [17]. Finally, the sample size of preterm neonates (N = 78) and large number of swallows (N = 3355) used is a strength of this study. The collection of a database of preterm swallowing sounds across facilities worldwide would help facilitate and refine the model performance for future application in clinical practice that is reflective of a majority of preterm neonates seen in special care nurseries.

## Conclusion

Swallowing sounds collected from digital cervical auscultation in preterm neonates can be accurately (94%) identified and segmented when using a transfer machine learning approach. Application of this model could support the development of a digital CA app to automatically classify swallow sounds and improve objectivity for CA use in clinical practice within special care nurseries.

## Supplementary Information

Below is the link to the electronic supplementary material.

Supplementary file1 (DOCX 823 kb)

Supplementary file2 (DOCX 28 kb)

## Data Availability

Sharing of the data for this study can be made available upon ethics clearance.
