# Supplementary figures and images for: Using Machine Learning for the Automated Segmentation and Detection of Swallows Obtained by Digital Cervical Auscultation in Preterm Neonates
Source: Dysphagia. 2025 Sep 12;41(1):275–84. doi: 10.1007/s00455-025-10879-3 (PMC12950097; doi:10.1007/s00455-025-10879-3)

Supplemental Figure 1. Example of a waveform plot from Adobe Audition (CS6 v5, Adobe Systems, USA).


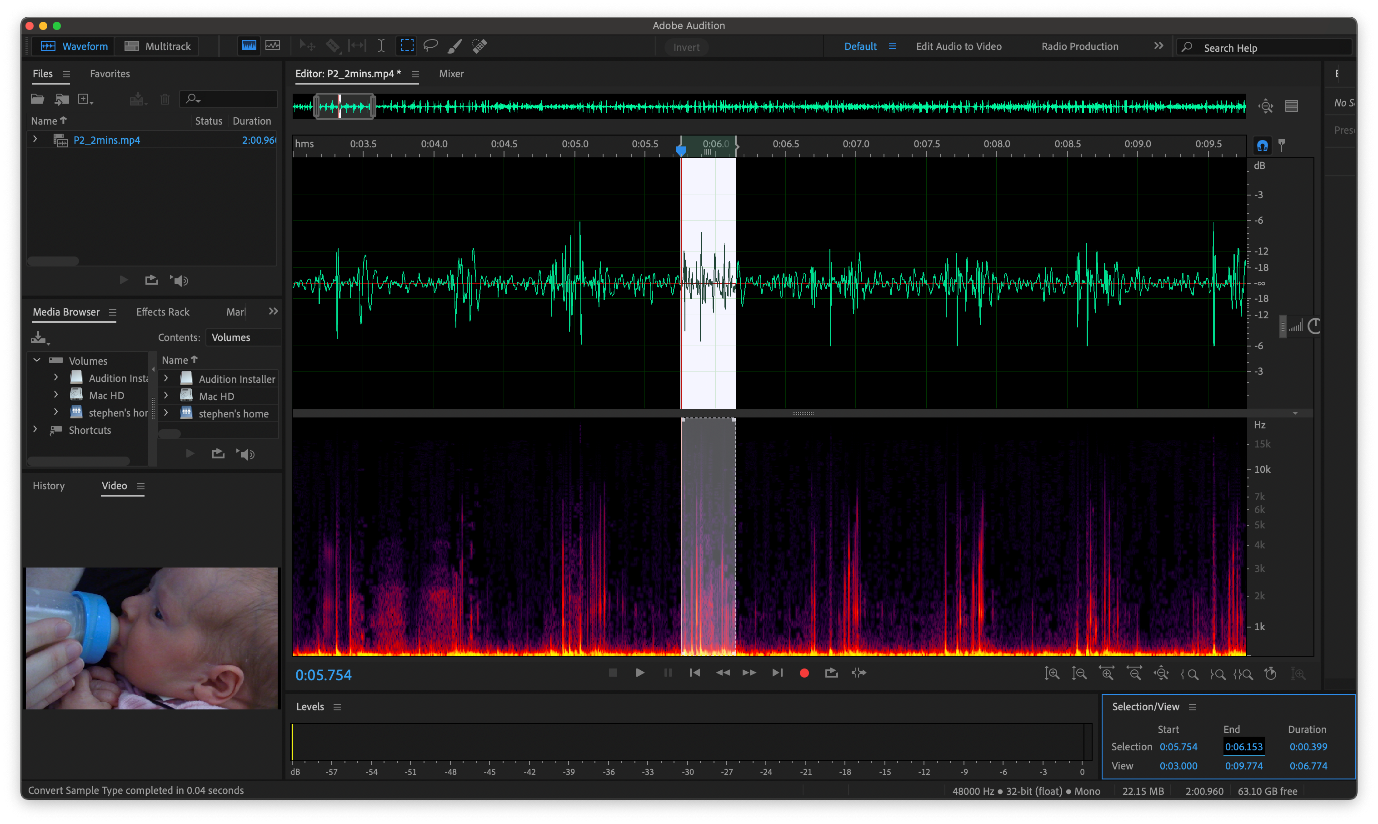

Supplement: Supplementary file 1 — Supplementary file1 (DOCX 823 kb) [file 455_2025_10879_MOESM1_ESM.docx]
