# Supplementary material for: Using Machine Learning for the Automated Segmentation and Detection of Swallows Obtained by Digital Cervical Auscultation in Preterm Neonates
Source: Dysphagia. 2025 Sep 12;41(1):275–84. doi: 10.1007/s00455-025-10879-3 (PMC12950097; doi:10.1007/s00455-025-10879-3)
Supplement: Supplementary file 2 — Supplementary file2 (DOCX 28 kb) [file 455_2025_10879_MOESM2_ESM.docx]

Supplemental Table 1. Glossary for digital signal processing and artificial intelligence terms

| Terminology | Definition |
| --- | --- |
| Activation function | Typically, a non-linear mathematical function that is applied on the output of each unit (or neuron) in a neural network in order to non-linearly map feature vectors in a way that enables the classes to be more easily separable. |
| Adam optimizer | A special optimization algorithm used in the training of neural network to find the best network parameters that minimize the desired objective or loss function, e.g. mean squared error between the model predictions and ground truths. |
| Classifier | A model or algorithm that is used to categorise data into different classes or categories, by analysing input data and assigning it to one of the predefined classes based on certain features or patterns. |
| Convolutional layer | A special type of layer used in convolutional neural networks (CNNs), where the input signal is convolved with the impulse response of a filter (also known as a kernel) |
| Decimation | The process of removing data samples in order to reduce the sampling frequency of a digital signal. |
| Deep learning | A subset of machine learning that involves neural networks with many layers that can model complex patterns in data by learning hierarchical representations. |
| Deep neural network (DNN) | A neural network architecture that contains three or more hidden layers. |
| Dynamic time-warping template matching | A method based on dynamic programming for measuring the similarity between two temporal sequences that may vary non-linearly in duration and speed. |
| Epoch | In the context of training neural networks, an epoch refers to one complete cycle through the entire training dataset. |
| Feature vectors | Vectors of numbers that are derived from a signal and contain information that is useful for classification tasks. |
| Finite impulse response (FIR) | A class of digital filter where the number of coefficients in its impulse response is finite. |
| Fully-connected neural network (FCNN) | Also known as a multiplayer perceptron (MLP), this is a supervised learning model for classification and regression that is biologically-inspired by the interconnection of neurons in the brain. |
| Gaussian mixture model (GMM) | A probabilistic maximum-likelihood parametric framework using a weighted mixture of Gaussian sources to model data. |
| Hidden markov model (HMM) | A probabilistic framework that models data as observations of non-observable states of some Markov process. |
| Interpolation | The process of reconstructing data samples between existing points to either convert from digital to analogue representation, or to increase the sampling frequency of a signal. This is usually performed by a specially designed lowpass filter. |
| Neural network (NN) | A generalised supervised machine learning model that is biologically-inspired by the interconnection of neurons in the brain. There are many different architectures, such as fully-connected neural networks, convolution neural networks, recurrent neural networks, etc. |
| log-Mel spectrogram | Time-frequency analysis of a signal where frequency has been warped according to the Mel-scale. |
| Machine learning | A branch of artificial intelligence that focuses on developing algorithms and statistical models that enable computers to learn from and make predictions or decisions based on data. |
| Mel frequency cepstrum coefficients (MFCC) | Handcrafted features used in previous generation automatic speech recognition systems that attempted to mimic the human auditory system. |
| Mel-scale filter | A filterbank that is spaced according the Mel-scale that is used to mimic the perception of sounds by the human auditory system. |
| Mel-scale warping | A non-linear frequency scale that correlates to perceptually equi-distant pitch. |
| Overfitting | An undesirable characteristic of a machine learning model, where during training, the model has excessively learned the unique specifics of the limited training data set but performs poorly on unseen data. |
| Rectified linear unit (ReLU) | A non-linear activation function used in hidden layer units of a neural network, where negative input values are mapped to zero, while positive input values pass through untouched. |
| Regularization | A technique used in the training of machine learning models to mitigate the effects of overfitting. An example is L2 regularization, which penalizes large model parameters. |
| Segmentation | The task of dividing a signal into regions that belong to the same class or category. For manual segmentation, a human determines the start and end points of each region; while in automatic segmentation, a machine performs the task using machine learning algorithms. |
| Sigmoid activation function | A monotonically-increasing activation function that non-linearly maps the entire set of real numbers to another set of real numbers that occur between zero and one. |
| Softmax layer | A special output layer used in neural network that uses a non-linear mathematical function to normalise a data vector such that each element represents a probability. That is, each element is a number between zero and one, and the sum of the elements in the vector equal one. |
| Support vector machine (SVM) | A supervised machine learning model for classification that finds the optimum decision surface by maximizing the margin, i.e. minimum distance between support vectors and the decision surface. |
| Transfer learning | A machine learning method where a model developed for one task is recycled as the starting point for a model on a second similar task. It is often used for tasks which do not have enough data to train a deep neural network. |
| YAMNet | Also known as “Yet another Audio Mobilenet Network”, this is a publicly-available deep neural network model for audio classification that was pretrained by Google to predict 521 audio event classes from the AudioSet corpus, which comprised audio from short YouTube clips |
| Zero-crossing rate (ZCR) | A feature used in previous generation automatic speech recognition system that measures the rate of sign changes in a signal. |
